# Singular Case of Spontaneous Recovery

**Published:** 1809-09

**Authors:** Joseph Jevans

**Affiliations:** Bloxham


### *To the Editors of the Medical and Physical Journal.*

Gentlemen,

THERE is now living at Dunstow, in Oxfordshire, one William Goodwin, the son of George Goodwin, a carpenter, who, when he was about 12 years of age, was kicked kicked by a horse a little below his stomach, rather towards the right side. He soon after became out of health, and complained of a pain in his bowels, which swelled very much. He had a fever and strong fits, in which he sometimes continued an hour or more; was delirious, and confined to his bed for half a year; and when he left it, was carried to and from it like a child. During this time he lost by degrees the use of his speech, his left arm, both his legs, and his appetite for his usual food and malt liquor; eating nothing but raw cheese, raw peas, and fruit of all kinds, whether native or foreign; but he principally subsisted on raw cheese, raw peas, and drank water. When he became a little better he moved about by means of a pair of crutches. But though he walked down stairs and steps in a natural way, he could only ascend them by walking backwards. After continuing in this state for seven or eight years, he went to the farm-houses in the village, to do such work as a cripple could perform; for by this time he had recovered the use of his left arm, and was tolerable healthy and cheerful.

In this improved state of health he continued for five or six years more; and about two years ago, laid aside, by very slow degrees, both his crutches. As his strength returned, his appetite for common food returned also. First he wished for a very small bit of cake or pudding, which though he ate it, did not agree with him; it was the same with other kinds of food, till he could eat all sorts of them; still, however, he continued dumb.

About a year ago, he was again so ill, that his life was thought to be in danger. During this last affliction, he one night said to a boy that slept with him, "I can speak." The youth being much surprised, informed some other branches of the family, who got up, and found it to be true. From that time his speech returned by hasty degrees, and in a few weeks his health was also restored. At this time he appears, to the admiration of his neighbours, perfectly recovered, save that his memory seems in some instances impaired; and I suspect, from his countenance, &c. that all his mental powers have not yet regained their proper vigour. He goes to the work of a day labourer, and is a member of a volunteer corps in that neighbourhood.

Almost every fact in this account I received this day from the young man himself, and his mother, at their house in Dunstow; and the substance of it I had received ceived several times before from their neighbours. I hope some medical gentleman will favour us with a philosophical solution of these phenomena.

I am, &c.

JOSEPH JEVANS.

